# Comparative efficacy of acupuncture point stimulation treatments for dialysis patients with uremic pruritus: a systematic review and network meta-analysis

**DOI:** 10.3389/fneur.2024.1342788

**Published:** 2024-03-22

**Authors:** Po-Hsuan Lu, Hui-En Chuo, Ling-Ya Chiu, Chien-Cheng Lai, Jen-Yu Wang, Ping-Hsun Lu

**Affiliations:** ^1^Department of Dermatology, MacKay Memorial Hospital, Taipei, Taiwan; ^2^Department of Medicine, MacKay Medical College, New Taipei City, Taiwan; ^3^Department of Medical Education, MacKay Memorial Hospital, Taipei, Taiwan; ^4^School of Post-Baccalaureate Chinese Medicine, Tzu Chi University, Hualien, Taiwan; ^5^Department of Chinese Medicine, Taipei Tzu Chi Hospital, Buddhist Tzu Chi Medical Foundation, New Taipei City, Taiwan

**Keywords:** uremic pruritus, acupuncture, acupoint injection, auricular acupressure, acupoint massage

## Abstract

**Background:**

Uremic pruritus (UP) is a common complication of chronic kidney disease that causes sleep disturbances and increases all-cause mortality. Currently, the first-line medications for UP exhibit inadequate pruritus control with adverse effects. Various acupuncture point stimulation treatments (APSTs) have been shown to be effective as adjuvant therapies in UP, and a network meta-analysis can offer relative efficacy estimates for treatments for which head-to-head studies have not been performed.

**Methods:**

We conducted a random-effects network meta-analysis on a consistency model to compare the different APSTs for UP. The primary outcomes were the mean visual analog scale (VAS) score and effectiveness rate (ER).

**Results:**

The network meta-analysis retrieved 27 randomized controlled trials involving 1969 patients. Compared with conventional treatment alone, combination treatment with acupuncture (mean difference, −2.63; 95% confidence interval, −3.71 to −1.55) was the most effective intervention in decreasing VAS scores, followed by acupoint injection and massage (mean difference, −2.04; 95% confidence interval, −3.96 to −0.12). In terms of the ER, conventional treatment with acupuncture and hemoperfusion (risk ratio, 14.87; 95% confidence interval, 2.18 to 101.53) was superior to other therapeutic combinations. Considering the VAS score and ER, combination treatment with acupoint injection and massage showed benefits in treating UP.

**Conclusion:**

Our network meta-analysis provided relative efficacy data for choosing the optimal adjuvant treatment for UP. Combined treatment with acupuncture was more effective than conventional treatment only and was the most promising intervention for treating UP.

**Systematic review registration**: PROSPERO (CRD42023425739: https://www.crd.york.ac.uk/prospero/display_record.php?ID=CRD42023425739).

## Introduction

Uremic pruritus (UP) is a troublesome complication of chronic kidney disease (CKD) with a prevalence ranging from 18 to 80% (1). Approximately 40% of patients with UP suffer from a moderate to severe itching with an inconsistent distribution and duration (2). Patients with UP experience sleep disturbances, depressive symptoms, impaired quality of life, an increased risk of infection, and even increased mortality (3, 4). The pathophysiology of UP is unclear and intricate; it involves skin changes, toxin deposition, immune dysregulation, metabolic changes, neuropathy, and an opioid imbalance (4, 5). Recent studies have challenged the role of the metabolism of minerals in the pathogenesis of UP; in contrast, many previous studies have focused on the inflammasome and nonhistaminergic pruritogens (4, 5). Antihistamines, gabapentin, and opioid-receptor modulators are the current treatments for UP, but these show variable therapeutic responses and potential side effects in patients with CKD (4, 6). Many adjuvant therapies including phototherapy, acupuncture, diet supplement, and sodium thiosulphate injection have recently been reported (6–8).

Studies on acupuncture point stimulation treatments (APSTs) for UP have shown efficacy in itch control (9–11). However, APSTs include various techniques: acupuncture, auricular acupressure (AA), acupoint injection (AI), acupoint massage (AM), acupoint sticking therapy (AST), acupoint infrared radiation (AIR), and acupoint transcutaneous electrical stimulation (ATENS) (12). The exact mechanism of action of acupuncture to treat UP remains poorly understood. Previous studies have found that the acupoints on the meridians are rich in high-density nerve endings (13). By stimulating the numerous membrane receptors on the peripheral nerves in different ways (i.e., electronic, chemical, or mechanical stimulation), acupuncture affects the regulation of cytokines, neuroimmunity, and the degranulation of mast cells (13). To date, no studies have compared the clinical efficacy of various APST techniques for treating UP. Therefore, to identify the effectiveness of different APSTs for UP, we performed a network meta-analysis to offer relative efficacy estimates for interventions that lack head-to-head studies.

## Methods

### Literature search

We searched seven major databases including PubMed, Embase, CINAH, the Cochrane Library, Wanfang, Chinese National Knowledge Infrastructure, and the Airiti Library from their inception to September 29, 2022, without language restriction. The search string used was based on MeSH and Emtree search headings: acupuncture (including acupuncture OR acupressure OR Shiatsu OR Zhi Ya OR Chih Ya OR Shiatzu OR auricular acupuncture OR ear acupuncture OR auricular acupressure OR ear acupressure OR auricular therapy OR auriculotherapy OR auricular needle OR otopoint OR otoneedle OR auriculoacupuncture OR otopuncture OR acupressure point OR acupoints OR Tui Na), pruritus, chronic kidney disease, dialysis, uremia. We also searched for free text words and word combinations containing the above mentioned terms (Supplementary Table S1). In addition to performing systematic searches of relevant databases, we conducted manual searches of the reference sections of the retrieved papers and contacted leading experts in the field to identify additional research studies. Furthermore, we examined unpublished studies available in the ClinicalTrials.gov registry^1^. This research is registered with PROSPERO (CRD42023425739). To ensure transparency and adherence to best practices, the reporting of this systematic review followed the guidelines outlined in the Preferred Reporting Items for Systematic Reviews and Meta-Analyses (PRISMA) extended statement for network meta-analysis.

### Study selection

Randomized controlled trials (RCTs) were selected based on the following inclusion criteria: (1) inclusion of dialysis patients with UP, (2) utilization of APST, and (3) availability of quantitative data regarding itching severity. Articles were excluded based on the following criteria: (1) non-randomized study design, (2) absence of a UP diagnosis, and (3) inclusion of control groups receiving additional treatments such as oral Chinese herbal medicine, herbal baths, charcoal tablets, or antihistamines. In cases in which data were either missing or in raw form, we contacted the authors via email for clarification. In situations in which multiple articles presented overlapping data, we excluded duplicate articles and selected those with larger population sizes.

### Data extraction and risk of bias assessment

According to the above mentioned inclusion and exclusion criteria, two reviewers (Hui-En Chuo and Ping-Hsun Lu) evaluated the initially selected studies for eligibility for the network meta-analysis independently. The comments of the two reviewers were recorded and compared. Any disagreements were submitted and resolved by a third reviewer later. For each selected study, the following information was listed: first author, publication year, number of patients, patient ages, intervention modes of acupuncture, concomitant treatment, severity of pruritus, inspection data, effectiveness rate (ER), and acupoint.

Two reviewers independently performed a quality assessment of each study using the Risk of Bias 2 tool recommended by the Cochrane Collaboration (14). The assessment tool addresses five domains to evaluate the methodological quality of the included RCTs. Any difference of opinions between the reviewers was determined by a third reviewer.

### Outcome measurement

Two outcomes were extracted and analyzed: visual analog scale (VAS) scores and ER of UP in dialysis patients. Higher VAS scores (0–10 points on each scale) indicate more severe pruritus. The definition of the ER is the number of patients with resolved pruritus or improvement in symptoms among the total patients which complain from UP.

### Statistical analysis and software

To compare the groups, continuous consequences, such as VAS scores, were measured as weighted mean differences, and the ER was measured using dichotomous outcomes. We used the network package of STATA (version MP 17.0, StataCorp, College Station, TX, United States) to perform the statistical analyses as well as to generate the figures and tables (15). The surface under the cumulative ranking curve (SUCRA) is an index that ranges between 0 and 1; a larger value is associated with a better treatment response. Funnel plots were generated to detect publication bias. RevMan 5.4 (Cochrane Collaboration, Copenhagen, Denmark) was applied to evaluate the risk of bias graph and summary.

## Results

### Characteristics of the included studies

**Figure 1:**
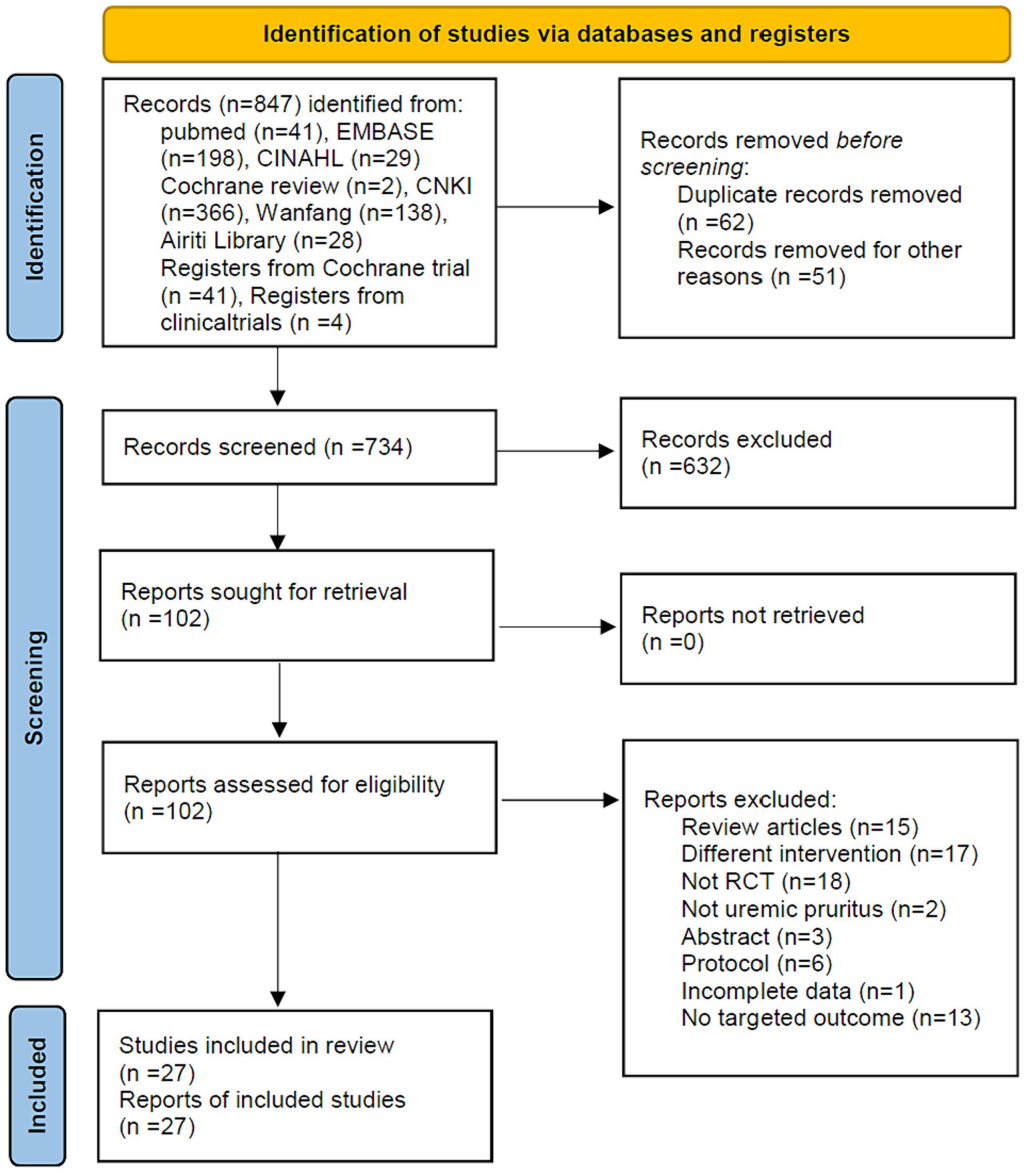
PRISMA 2020 flow diagram.

To evaluate the effect of APSTs for UP, RCTs were identified according to the PRISMA flowchart process (Figure 1). We identified 847 articles from electronic databases and excluded 632 articles based on the titles and abstracts. A total of 102 full-text articles were reviewed, and 75 of these articles were excluded for the following reasons: 15 studies were review articles, 17 studies involved different interventions, 18 studies were not RCTs, 2 studies did not involve patients with UP, 3 studies had abstracts only, 6 studies were protocols, 1 study provided incomplete data, and 13 studies did not report the target outcome. We synthesized the remaining 27 articles qualitatively and quantitatively.

Table 1 lists the characteristics of the retrieved RCTs. The included studies were published between 2004 and 2021 and had a total of 1,969 participants. The sample size of the included RCTs ranged from26–150 participants. All acupuncture interventions are combined with conventional therapy (dialysis for electrolyte balance, blood pressure, and body fluid maintenance). In studies following APSTs in patients with UP on dialysis, 11 involved acupuncture, nine involved AA, two evaluated patients were treated with AI and acupuncture (AI + A), and the remaining seven examined patients were treated with the following: acupoint far infrared (AFIR), AI, AI combined with AM (AI + AM), AIR, AM, AST, or ATENS. The acupoints used for each acupuncture therapy are listed in Supplementary Table S2.

**Table 1:** League table comparing different APSTs in terms of overall ER and VAS score.

(A) Overall ER								
**Tx + A + HP**	0.17 (0.02, 1.28)	0.09 (0.01, 0.63)^*^	0.08 (0.01, 0.58)^*^	0.09 (0.01, 0.62)^*^	0.08 (0.01, 0.56)^*^	0.07 (0.01, 0.49)^*^	0.07 (0.01, 0.49)^*^	0.07 (0.01, 0.46)^*^
5.95 (0.78, 45.18)	**Tx + AA + ND**	0.54 (0.27, 1.10)	0.50 (0.26, 0.97)^*^	0.52 (0.24, 1.12)	0.49 (0.25, 0.94)^*^	0.43 (0.22, 0.82)^*^	0.42 (0.21, 0.83)^*^	0.40 (0.21, 0.77)^*^
11.01 (1.58, 76.83)^*^	1.85 (0.91, 3.77)	**Tx + AI + AM**	0.93 (0.68, 1.28)	0.96 (0.58, 1.60)	0.90 (0.66, 1.23)	0.79 (0.58, 1.08)	0.78 (0.55, 1.11)	0.74 (0.56, 0.99)^*^
11.84 (1.73, 81.19)^*^	1.99 (1.03, 3.86)^*^	1.07 (0.78, 1.47)	**Tx + A**	1.03 (0.67, 1.60)	0.97 (0.82, 1.16)	0.85 (0.72, 1.00)	0.84 (0.65, 1.07)	0.80 (0.70, 0.91)^*^
11.44 (1.60, 81.63)^*^	1.92 (0.89, 4.15)	1.04 (0.63, 1.72)	0.97 (0.63, 1.49)	**Tx + A + HDF**	0.94 (0.61, 1.45)	0.82 (0.53, 1.26)	0.81 (0.51, 1.29)	0.77 (0.51, 1.16)
12.18 (1.78, 83.45)^*^	2.05 (1.06, 3.96)^*^	1.11 (0.81, 1.51)	1.03 (0.86, 1.22)	1.06 (0.69, 1.64)	**Tx + AA**	0.87 (0.73, 1.03)	0.86 (0.68, 1.10)	0.82 (0.73, 0.92)^*^
13.99 (2.04, 95.92)^*^	2.35 (1.22, 4.55)^*^	1.27 (0.93, 1.74)	1.18 (1.00, 1.40)	1.22 (0.79, 1.89)	1.15 (0.97, 1.36)	**Tx + AAI**	0.99 (0.78, 1.27)	0.94 (0.83, 1.07)
14.13 (2.05, 97.59)^*^	2.38 (1.20, 4.70)^*^	1.28 (0.90, 1.83)	1.19 (0.93, 1.53)	1.24 (0.78, 1.97)	1.16 (0.91, 1.48)	1.01 (0.79, 1.29)	**Tx + AA + HP**	0.95 (0.77, 1.17)
14.87 (2.18, 101.53)^*^	2.50 (1.31, 4.78)^*^	1.35 (1.01, 1.80)^*^	1.26 (1.10, 1.43)^*^	1.30 (0.86, 1.97)	1.22 (1.09, 1.37)^*^	1.06 (0.94, 1.20)	1.05 (0.85, 1.30)	**Tx**

(B) VAS											
**Tx + A**	0.59 (−1.61, 2.79)	0.67 (−1.67, 3.01)	0.78 (−1.83, 3.39)	0.91 (−1.46, 3.28)	1.08 (−0.37, 2.52)	1.60 (−0.55, 3.75)	2.13 (−0.31, 4.57)	2.08 (−0.12, 4.28)	2.64 (0.45, 4.84)^*^	2.78 (0.32, 5.25)^*^	2.63 (1.55, 3.71)^*^
−0.59 (−2.79, 1.61)^*^	**Tx + AI + AM**	0.08 (−2.75, 2.91)	0.19 (−2.86, 3.24)	0.32 (−2.53, 3.17)	0.48 (−1.66, 2.63)	1.01 (−1.66, 3.68)	1.54 (−1.37, 4.45)	1.49 (−1.22, 4.20)	2.05 (−0.66, 4.76)	2.19 (−0.74, 5.12)	2.04 (0.12, 3.96)^*^
−0.67 (−3.01, 1.67)	−0.08 (−2.91, 2.75)	**Tx + ATENS**	0.11 (−3.05, 3.27)	0.24 (−1.95, 2.43)	0.40 (−1.88, 2.69)	0.93 (−1.86, 3.72)	1.46 (−1.56, 4.48)	1.41 (−1.41, 4.23)	1.97 (−0.85, 4.79)	2.11 (−0.93, 5.15)	1.96 (−0.12, 4.04)
−0.78 (−3.39, 1.83)	−0.19 (−3.24, 2.86)	−0.11 (−3.27, 3.05)	**Tx + AIR**	0.13 (−3.05, 3.31)	0.29 (−2.27, 2.86)	0.82 (−2.20, 3.84)	1.35 (−1.88, 4.58)	1.30 (−1.75, 4.35)	1.86 (−1.19, 4.91)	2.00 (−1.25, 5.25)	1.85 (−0.53, 4.23)
−0.91 (−3.28, 1.46)	−0.32 (−3.17, 2.53)	−0.24 (−2.43, 1.95)	−0.13 (−3.31, 3.05)	**Tx + AM**	0.16 (−2.15, 2.48)	0.69 (−2.12, 3.50)	1.22 (−1.82, 4.26)	1.17 (−1.68, 4.02)	1.73 (−1.11, 4.57)	1.87 (−1.19, 4.93)	1.72 (−0.39, 3.83)
−1.08 (−2.52, 0.37)	−0.48 (−2.63, 1.66)	−0.40 (−2.69, 1.88)	−0.29 (−2.86, 2.27)	−0.16 (−2.48, 2.15)	**Tx + AA**	0.53 (−1.57, 2.62)	1.06 (−1.33, 3.44)	1.01 (−1.14, 3.15)	1.57 (−0.57, 3.70)	1.71 (−0.71, 4.12)	1.56 (0.60, 2.52)^*^
−1.60 (−3.75, 0.55)	−1.01 (−3.68, 1.66)	−0.93 (−3.72, 1.86)	−0.82 (−3.84, 2.20)	−0.69 (−3.50, 2.12)	−0.53 (−2.62, 1.57)	**Tx + AI**	0.53 (−2.34, 3.40)	0.48 (−2.19, 3.15)	1.04 (−1.63, 3.71)	1.18 (−1.71, 4.07)	1.03 (−0.83, 2.89)
−2.13 (−4.57, 0.31)	−1.54 (−4.45, 1.37)	−1.46 (−4.48, 1.56)	−1.35 (−4.58, 1.88)	−1.22 (−4.26, 1.82)	−1.06 (−3.44, 1.33)	−0.53 (−3.40, 2.34)	**Tx + AST + HF**	−0.05 (−2.96, 2.86)	0.51 (−2.39, 3.41)	0.65 (−2.46, 3.76)	0.50 (−1.69, 2.69)
−2.08 (−4.28, 0.12)	−1.49 (−4.20, 1.22)	−1.41 (−4.23, 1.41)	−1.30 (−4.35, 1.75)	−1.17 (−4.02, 1.68)	−1.01 (−3.15, 1.14)	−0.48 (−3.15, 2.19)	0.05 (−2.86, 2.96)	**Tx + AST + HPF**	0.56 (−2.15, 3.27)	0.70 (−2.23, 3.63)	0.55 (−1.36, 2.46)
−2.64 (−4.84, −0.45)^*^	−2.05 (−4.76, 0.66)	−1.97 (−4.79, 0.85)	−1.86 (−4.91, 1.19)	−1.73 (−4.57, 1.11)	−1.57 (−3.70, 0.57)	−1.04 (−3.71, 1.63)	−0.51 (−3.41, 2.39)	−0.56 (−3.27, 2.15)	**Tx + AFIR**	0.14 (−2.79, 3.07)	−0.01 (−1.92, 1.90)
−2.78 (−5.25, −0.32)^*^	−2.19 (−5.12, 0.74)	−2.11 (−5.15, 0.93)	−2.00 (−5.25, 1.25)	−1.87 (−4.93, 1.19)	−1.71 (−4.12, 0.71)	−1.18 (−4.07, 1.71)	−0.65 (−3.76, 2.46)	−0.70 (−3.63, 2.23)	−0.14 (−3.07, 2.79)	**Tx + AST**	−0.15 (−2.37, 2.07)
−2.63 (−3.71, −1.55)^*^	−2.04 (−3.96, −0.12)^*^	−1.96 (−4.04, 0.12)	−1.85 (−4.23, 0.53)	−1.72 (−3.83, 0.39)	−1.56 (−2.52, −0.60)^*^	−1.03 (−2.89, 0.83)	−0.50 (−2.69, 1.69)	−0.55 (−2.46, 1.36)	0.01 (−1.90, 1.92)	0.15 (−2.07, 2.37)	**Tx**

The relative risks, mean differences, and 95% confidence intervals are presented. To interpret the findings, read the columns from left to right, as they represent decreasing treatment efficacy. The intervention in the top left corner demonstrates the best outcome in the network meta-analysis. Statistically significant comparisons are indicated by (^*^). A, acupuncture; AA, aricular acupressure; AFIR, acupoint far infrared; AI, acupoint injection; AIR, acupoint infrared; AM, acupoint massage; AST, acupoint sticking therapy; ATENS, acupoint transcutaneous electrical nerve stimulation HF, hemofiltration; HDF, hemodiafiltration; HP, hemoperfusion; ND, nocturnal dialysis; Tx, conventional treatment.

### Risk of bias assessment

The results of the risk of bias assessment charts for the included studies are shown in Supplementary Figures S1A,B. All the included trials were randomly allocated, and seven studies provided information about the hidden allocation sequence. Twelve studies described random methods (seven studies used tables of random numbers, three studies used random number generators, and one study used drawing of lots, and one study used chart numbers). Eight studies did not use intention-to-treat analysis, and the loss-to-follow-up rate of eight studies was greater than 5%. There were eight studies with lost outcome data >5%; none had any evidence to support the consistency of the missing data analysis results. Four studies reported different reasons for missing data, and the others did not report the reasons for missing data. Only two studies were double-blinded, and 1 study was single-blinded. The overall bias and outcome measurement domains exhibited a high risk of bias because most of the studies were not double-blinded and self-assessed itch scores are subjective outcomes. All outcome data were available from the included trials.

### Overall effectiveness rate

**Figure 2:**
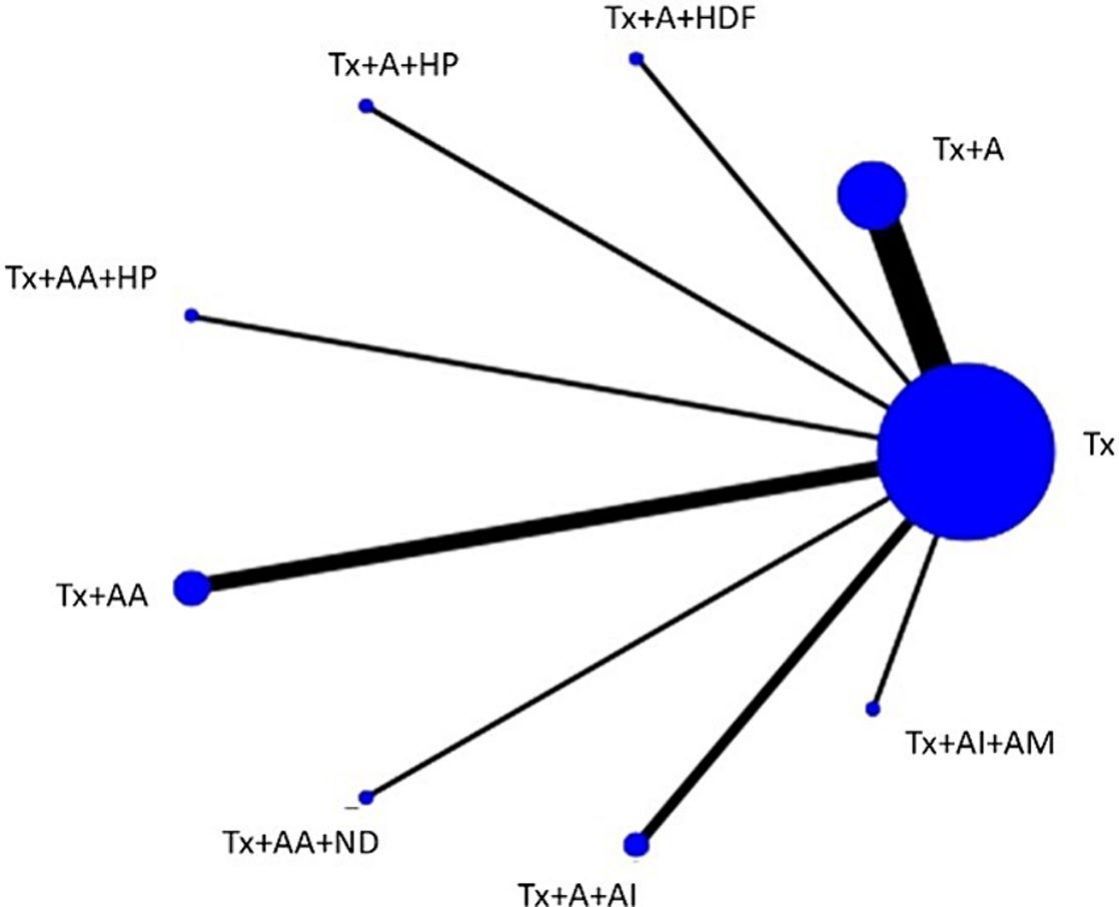
Network plot of the network meta-analysis on ERs every node represents a specific treatment, where the size of each node reflects the number of subjects involved, and the thickness of the lines corresponds to the number of RCTs providing comparative data. A, acupuncture; AA, aricular acupressure; AI, acupoint injection; AM, acupoint massage; HDF, hemodiafiltration; HP, hemoperfusion; ND, nocturnal dialysis; Tx, conventional treatment.

**Figure 3:**
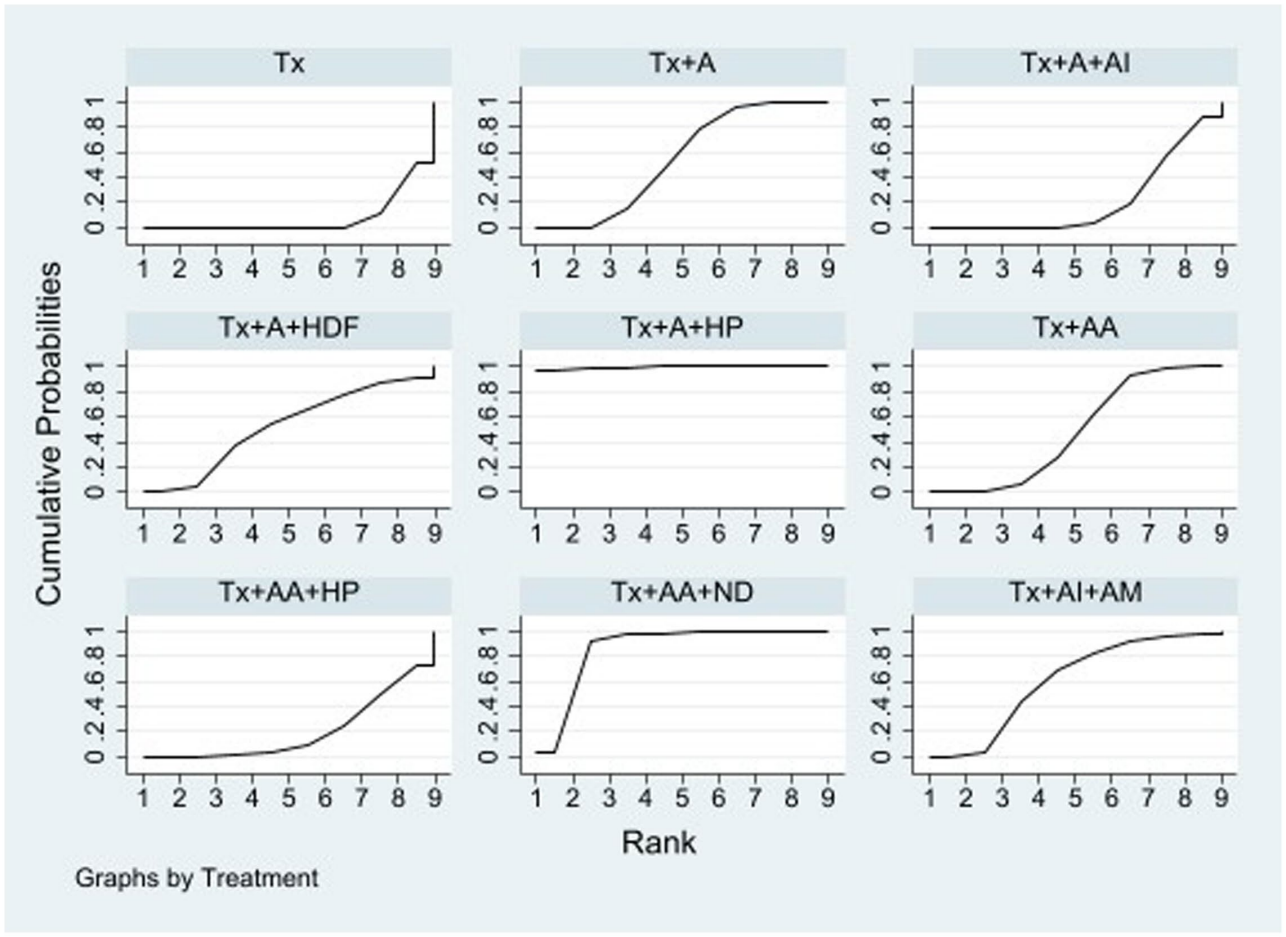
SUCRA of the network meta-analysis on ERs surface under the cumulative ranking curve. The overall ranking of each treatment is determined by the SUCRA. A greater area under the curve indicates a higher ranking, which signifies a superior treatment in the network meta-analysis. A, acupuncture; AA, aricular acupressure; AI, acupoint injection; AM, acupoint massage; HDF, hemodiafiltration; HP, hemoperfusion; ND, nocturnal dialysis; Tx, conventional treatment.

Sixteen studies including eight types of APST combinations in the network meta-analysis reported overall ERs. Six studies involved acupuncture, three involved AA, two involved AI + A, and the other involved the following different acupuncture treatment combinations: acupuncture with hemodiafiltration (A + HDF), acupuncture with hemoperfusion (A + HP), auricular acupressure with hemoperfusion (AA + HP), auricular acupressure with nocturnal dialysis 3 times per week (AA + ND), and AI + AM. For different APST combinations, the network graph (Figure 2) shows that the most publications evaluated traditional treatments, followed by APST, while A + HDF was the subject of few publications. Most trials reported comparisons between usual treatment (Tx) and acupuncture. Table 1 presents a comprehensive overview of the acupuncture treatment combinations that led to increased ERs through a direct comparison. In dialysis patients, the addition of different acupuncture treatment combinations to Tx, including Tx + A + HP, Tx + AA+ND, Tx + AI+AM, Tx + A, and Tx + AA, demonstrated a significant increase in the ERs of UP treatment compared with Tx alone. The treatment of Tx + A + HP (risk ratio (RR), 14.87; 95% confidence interval (CI), 2.18 to 101.53) was the most effective in increasing the ER, followed by Tx + AA+ND, Tx + AI+AM, and Tx + A, which were superior to Tx + AA (Table 1A). The overall ranking of each treatment can be visualized through the SUCRA. In Figure 3 and Table 2A, the descending order of SUCRA rankings for the ER of APST combinations in dialysis patients with UP are as follows: Tx + A + HP (SUCRA = 99.1), Tx + AA + ND (SUCRA = 86.3), Tx + AI + AM (SUCRA = 60.5), Tx + A (SUCRA = 54.2), Tx + A + HDF (SUCRA = 52.2), Tx + AA (SUCRA = 48.5), Tx + A + AI (SUCRA = 21.1), Tx + AA + HP (SUCRA = 20.3), and Tx (SUCRA = 7.8).

**Table 2:** SUCRA ranking of overall ER and VAS score in APSTs in dialysis patients.

(A) Overall ER			
Treatment	SUCRA	Pr Best	Mean Rank
Tx	7.8	0.0	8.4
Tx + A	54.2	0.0	4.7
Tx + A + HDF	52.2	0.0	4.8
Tx + A + HP	99.1	96.2	1.1
Tx + AA+HP	20.3	0.0	7.4
Tx + AA	48.5	0.0	5.1
Tx + AA+ND	86.3	3.8	2.1
Tx + A + AI	21.1	0.0	7.3
Tx + AI+AM	60.5	0.0	4.2

(B) Visual analogue scale			
Treatment	SUCRA	Pr Best	Mean Rank
Tx	17.9	0.0	10.0
Tx + A	88.0	34.9	2.3
Tx + AA	61.8	0.9	5.2
Tx + AFIR	22.6	0.3	9.5
Tx + AI	47.4	2.1	6.8
Tx + AI + AM	72.4	18.1	4.0
Tx + AIR	66.9	17.4	4.6
Tx + AST	20.7	0.5	9.7
Tx + AST + HPF	34.5	0.9	8.2
Tx + AST + HF	34.9	1.3	8.2
Tx + AM	63.1	9.4	5.1
Tx + ATENS	69.9	14.2	4.3

A, acupuncture; AA, aricular acupressure; AFIR, acupoint far infrared; AI, acupoint injection; AIR, acupoint infrared; AM, acupoint massage; AST, acupoint sticking therapy; ATENS, acupoint transcutaneous electrical nerve stimulation; HF, hemofiltration; HDF, hemodiafiltration; HP, hemoperfusion; ND, nocturnal dialysis; SUCRA, surface under the cumulative ranking curve; Tx, conventional treatment.

### Visual analog scale

**Figure 4:**
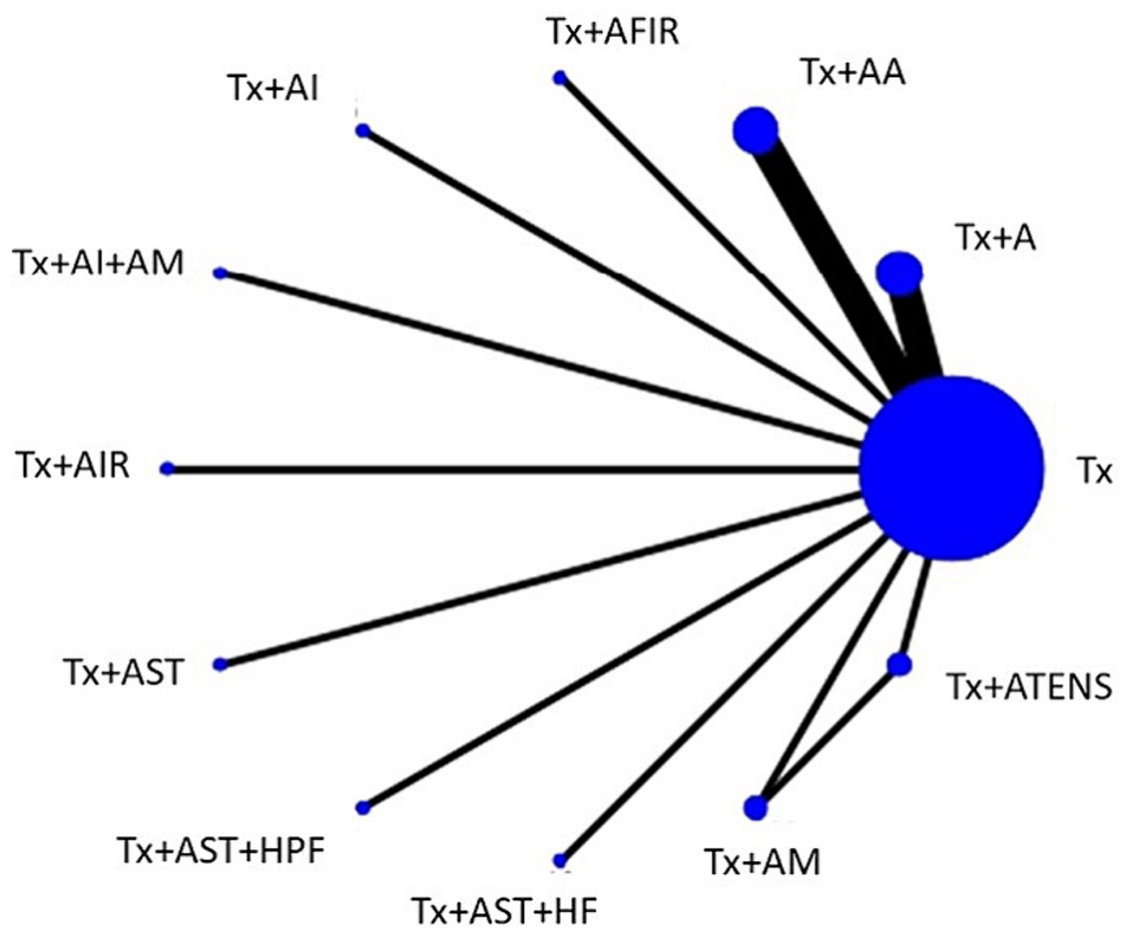
Network plot of the network meta-analysis for VAS scores. Every node represents a specific treatment, where the size of each node reflects the number of subjects involved, and the thickness of the lines corresponds to the number of RCTs providing comparative data. A, acupuncture; AA, aricular acupressure; AFIR, acupoint far infrared; AI, acupoint injection; AIR, acupoint infrared; AM, acupoint massage; AST, acupoint sticking therapy; ATENS, acupoint transcutaneous electrical nerve stimulation; HF, hemofiltration; HPF, hemodiafiltration; Tx, conventional treatment.

**Figure 5:**
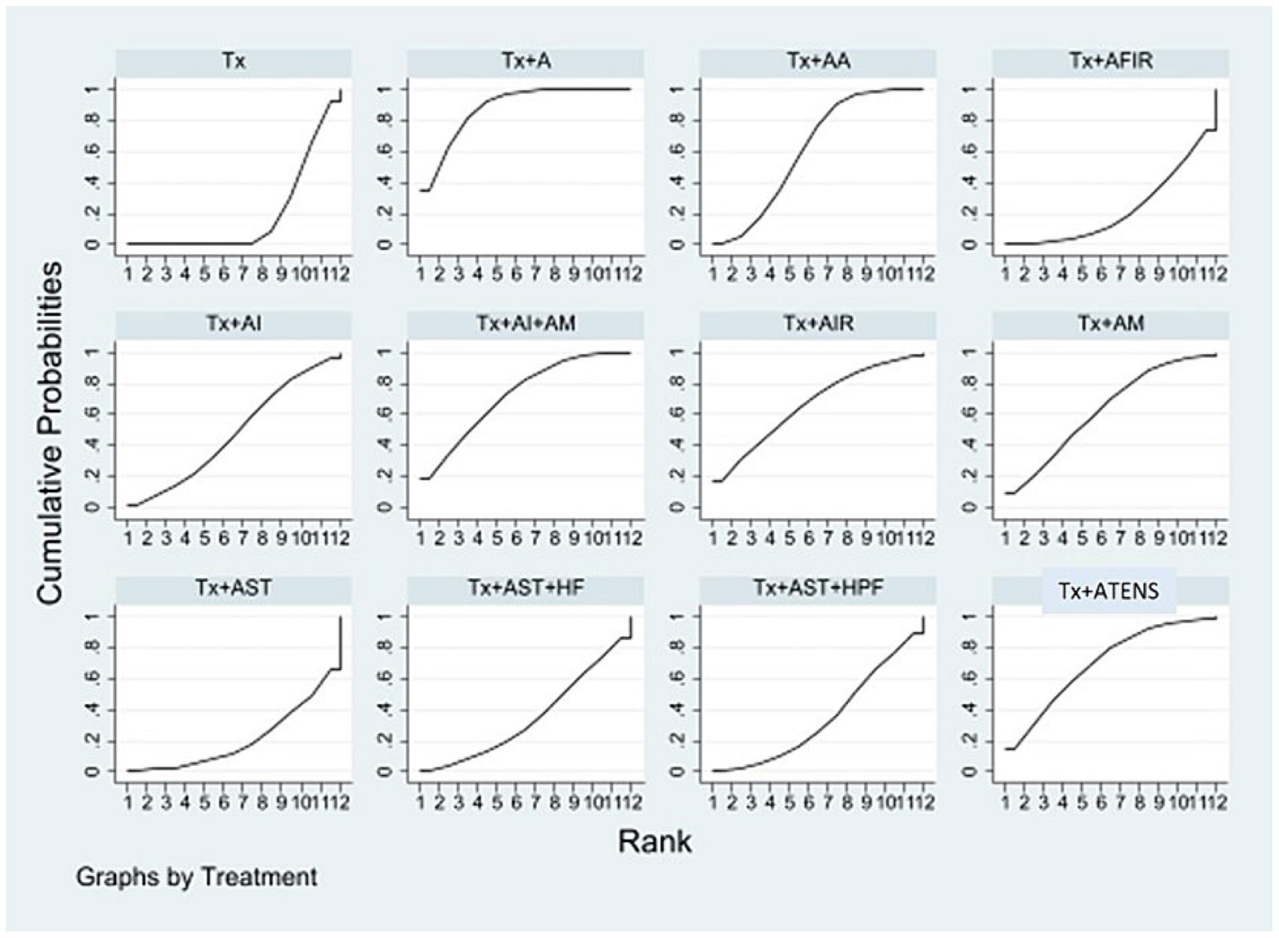
SUCRA of the network meta-analysis for VAS scores. Surface under the cumulative ranking curve. The overall ranking of each treatment is determined by the SUCRA. A greater area under the curve indicates a higher ranking, which signifies a superior treatment in the network meta-analysis. A, acupuncture; AA, aricular acupressure; AFIR, acupoint far infrared; AI, acupoint injection; AIR, acupoint infrared; AM, acupoint massage; AST, acupoint sticking therapy; ATENS, acupoint transcutaneous electrical nerve stimulation; HF, hemofiltration; HPF, hemodiafiltration; Tx, conventional treatment.

In the network meta-analysis, 16 studies reported VAS scores for 11 different combinations of APSTs. Among these, four studies used Tx + A, four used Tx + AA, and the remaining nine used various combinations, such as Tx + AFIR, Tx + AI, Tx + AI+AM, Tx + AIR, Tx + AST, Tx + AST + HPF, Tx + AST + HF, Tx + AM, and Tx + ATENS. In the network plot (Figure 4), it is evident that studies involving Tx + A and Tx + AA had the most subjects, whereas studies involving Tx + AFIR, Tx + AI, Tx + AI + AM, Tx + AIR, Tx + AST, Tx + AST + HPF, Tx + AST + HF, Tx + AM, and Tx + ATENS had only one subject per study. The majority of the trials reported a comparison between Tx and Tx + A. Table 1B, which shows the league table, presents the outcomes of both direct and indirect comparisons of the efficacy of various APSTs in decreasing VAS scores. The results indicate that Tx + A, Tx + AI + AM, and Tx + AA were significantly more effective in reducing VAS scores among dialysis patients with UP than Tx. The most successful intervention in reducing VAS scores among the tested treatments was Tx + A, with a mean difference (MD) of −2.63 (95% CI = −3.71 to −1.55). Following Tx + A, Tx + AI + AM was the second most effective treatment, with an MD of −2.04 (95% CI = −3.96 to −0.12). The ability of these treatments to reduce VAS scores of UP in dialysis patients was ranked based on SUCRA scores (Figure 5 and Table 2B). In descending order, the rankings were as follows: Tx + A (SUCRA = 88.0), Tx + AI + AM (SUCRA = 72.4), Tx + ATENS (SUCRA = 69.9), Tx + AIR (SUCRA = 66.9), Tx + AM (SUCRA = 63.1), Tx + AA (SUCRA = 61.8), Tx + AI (SUCRA = 47.4), Tx + AST + HF (SUCRA = 34.9), Tx + AST + HPF (SUCRA = 34.5), Tx + AFIR (SUCRA = 22.6), Tx + AST (SUCRA = 20.7), and Tx (SUCRA = 17.9).

### Cluster ranking plot of different APSTs for up in dialysis patients

The predicted SUCRA values for ERs and VAS scores were utilized to generate a cluster ranking plot (Supplementary Figure S2). Data on both ERs and VAS scores were available for the three APSTs: Tx + A, Tx + AI + AM, and Tx + AA. According to the analysis, Tx + AI + AM was expected to have a greater impact on improving both ER and VAS scores than Tx + AA. In addition, Tx + A was identified as the most effective treatment for improving the ER.

### Publication bias

Supplementary Figure S3 displays the funnel plots that were generated to identify any publication bias in studies reporting ERs and VAS scores. The asymmetrical distributions observed in both funnel plots suggest the possibility of publication bias among the studies included in the analysis.

## Discussion

Our network meta-analysis retrieved 27 randomized controlled trials involving 1,684 patients showed that the treatment combination of Tx + A were found to be the most effective in improving the VAS scores. A possible target of acupuncture in skin is sensory cutaneous innervation, which is abundant in cases of neuropathic pruritus, prurigo nodularis, and UP (16, 17). However, a previous review, which retrieved only English literature, reported that the effectiveness of acupuncture in treating UP is controversial (11). Lu et al. recently conducted a systematic review that demonstrated the effectiveness of APST in reducing VAS scores of UP and biomarkers, such as blood urea nitrogen (BUN), parathyroid hormone (PTH), and histamine levels (18). The most commonly used acupoints are reported to be SP10, LI11, SP6, and ST36, suggesting that acupuncture could serve as a beneficial complementary treatment for alleviating UP (19). Moreover, the application of six weeks of acupuncture at LI11 in patients undergoing hemodialysis led to a decrease in the dimensions of the 5-D itch scale, specifically in terms of degree, duration, disability, and distribution (20). Chang et al. conducted an RCT demonstrating the efficacy of acupuncture at bilateral LI11, SP10, and SP6 in reducing itching symptoms among patients undergoing hemodialysis. In addition, the intervention showed a significant improvement in VAS and Duo scores, an increase in albumin indicators, and a decrease in immunoglobulin E indicators (21). In the mouse cheek model of pruritogen-induced acute itch and an MC903-induced atopic dermatitis model displaying serotonergic chronic itch, acupuncture at LI11 significantly improved skin inflammation and alleviated both acute and chronic serotonergic itch. These effects may be mediated through the blockade of serotonin 5-hydroxytryptamine 2 and 5-HT7 receptors (22). In a rat model of urticaria induced by anti-ovalbumin serum, acupuncture at LI11 and SP10 was found to inhibit type I hypersensitivity and mast cell degranulation. These effects may be attributed to the regulation of p-Lyn and p-Syk protein expression in the locus coeruleus skin tissue (23).

Our cluster ranking plot showed that the combination of Tx + AI + AM and Tx + AA were the most effective in improving ERs and reducing VAS scores. AI is a technique in which a syringe needle is used to inject drugs or substances directly into acupoints (24). Compared with traditional acupuncture needles, AI has been shown to achieve equivalent or increased plasma concentrations of drugs following injection at acupoints such as ST36 and the femoral vein using carbamyl b-methylcholine chloride (25). In addition, AI at SP6 as the trigger point has been found to significantly increase phylloquinone plasma concentration (26). Clinical studies have demonstrated that AI offers clear advantages and reliable therapeutic benefits in the management of UP and CKD (27, 28). AM involves applying pressure to meridian points using palms, fingertips, small beads, or specialized devices (11). This pressure on acupoints is thought to stimulate blood circulation and the secretion of neurotransmitters (29). Chen et al. conducted a study combining AM with AI in patients with UP and reported improved efficacy and VAS scores compared with a control group (28). AA, a traditional practice originating in ancient China, has been utilized to address specific organ functions and treat diseases (30). Nogier, a French physician, conducted systematic research that demonstrated the existence of functional relationships and dependencies between different regions of the ear and specific organs (31). AA was found to reduce both VAS scores and serum histamine levels in hemodialysis patients with UP in an RCT (32).

Tx + A + HP showed the greatest increase in the ER. Hemoperfusion is a blood purification technique that involves passing anticoagulated whole blood through an adsorbent particle-filled column or device (33). Furthermore, regular treatment with hemodialysis and hemoperfusion was found to be more effective than hemodialysis alone in eliminating middle and large-molecule uremic toxins that accumulate in the body (34). Hemodialysis and hemoperfusion can potentially improve UP, quality of life, and the survival rate of patients undergoing hemodialysis (34) as well as reduce beta2-microglobulin and PTH levels (35). The combination of acupuncture with hemodialysis and hemoperfusion demonstrated a better ER and greater reduction in Duo’s score than the group receiving hemodialysis alone for the treatment of UP (36). ND represents a significant approach to enhancing the effectiveness of dialysis and has been linked to numerous clinical advantages. Consistent evidence supports its positive impact on improving blood pressure regulation and managing phosphate and mineral metabolism (37).

Our network meta-analysis offers clinicians comparisons of different APSTs as adjunctive treatments for UP in dialysis patients, providing valuable clinical recommendations. Nonetheless, there were certain limitations to our study. First, incorporating multiple APSTs within a single trial may have resulted in small sample sizes, which could have led to inconsistent outcomes. For instance, when comparing Tx to Tx + A + HP, the latter exhibited a significantly higher ER but did not yield a significant reduction in VAS scores. Second, it is important to note that the majority of the trials included in the analysis were conducted within a single country, potentially limiting the generalizability of the findings to other populations or regions. Third, a significant proportion of the selected articles lacked long-term follow-up, which could impede a comprehensive understanding of the sustained effects of the treatments under investigation. Finally, variations in acupoint selection and treatment duration (which ranged from 10 days to over 4 months) may have influenced the outcome measures and should be considered when interpreting the results.

## Conclusion

In terms of the ER, Tx + A + HP outperformed other APSTs. As for reducing VAS scores, Tx + A was the most effective intervention. However, Tx + AI + AM was the most effective at improving both the ERs and VAS scores, followed by Tx + AA. Conducting head-to-head comparisons could be beneficial for shared decision-making to provide various adjunctive APST options for managing UP in dialysis patients.

## Data availability statement

The datasets presented in this study can be found in online repositories. The names of the repository/repositories and accession number(s) can be found in the article/Supplementary material (38–60).

## Author contributions

Po-HL: Writing – original draft. H-EC: Writing – original draft. L-YC: Writing – review & editing. C-CL: Writing – review & editing. J-YW: Writing – review & editing. Pi-HL: Writing – review & editing.

## Footnotes

^1^ http://clinicaltrials.gov/

## References

[1] Hu X Sang Y Yang M Chen X Tang W. Prevalence of chronic kidney disease-associated pruritus among adult dialysis patients: a meta-analysis of cross-sectional studies. Medicine. (2018) 97:e10633. doi: 10.1097/md.0000000000010633, PMID: 29794739 PMC6392722

[2] Pisoni RL Wikström B Elder SJ Akizawa T Asano Y Keen ML . Pruritus in haemodialysis patients: international results from the dialysis outcomes and practice patterns study (Dopps). Nephrol Dial Transplant. (2006) 21:3495–505. doi: 10.1093/ndt/gfl461, PMID: 16968725

[3] Kim D Pollock C. Epidemiology and burden of chronic kidney disease-associated pruritus. Clin Kidney J. (2021) 14:i1–7. doi: 10.1093/ckj/sfab142, PMID: 34987777 PMC8702817

[4] Verduzco HA Shirazian S. Ckd-associated pruritus: new insights into diagnosis, pathogenesis, and management. Kidney Int Rep. (2020) 5:1387–402. doi: 10.1016/j.ekir.2020.04.027 32954065 PMC7486142

[5] Schricker S Kimmel M. Unravelling the pathophysiology of chronic kidney disease-associated pruritus. Clin Kidney J. (2021) 14:i23–31. doi: 10.1093/ckj/sfab200, PMID: 34987780 PMC8702819

[6] Lipman ZM Paramasivam V Yosipovitch G Germain MJ. Clinical Management of Chronic Kidney Disease-Associated Pruritus: current treatment options and future approaches. Clin Kidney J. (2021) 14:i16–22. doi: 10.1093/ckj/sfab167, PMID: 34987779 PMC8702820

[7] Elhag S Rivas N Tejovath S Mustaffa N Deonarine N Abdullah Hashmi M . Chronic kidney disease-associated pruritus: a glance at novel and lesser-known treatments. Cureus. (2022) 14:e21127. doi: 10.7759/cureus.21127, PMID: 35036239 PMC8752116

[8] Lin C-H Lu P-H Yue C-T Hsieh P-C Lin Y-H Lan C-C . Chrysophanol triggers cell death via unfolded protein response and endoplasmic reticulum stress in Oral Cancer Fadu cells. Curr Topics Nutraceut Res. (2021) 19:64–8. doi: 10.37290/ctnr2641-452X.19:64-68

[9] Kim KH Lee MS Kim TH Kang JW Choi TY Lee JD. Acupuncture and related interventions for symptoms of chronic kidney disease. Cochrane Database Syst Rev. (2016) 2016:CD009440. doi: 10.1002/14651858.CD009440.pub2, PMID: 27349639 PMC8406453

[10] Zhang L Li Y Xiao X Shi Y Xu D Li N . Acupuncture for uremic pruritus: a systematic review and meta-analysis. J Pain Symptom Manag. (2022) 65:e51–62. doi: 10.1016/j.jpainsymman.2022.08.017 36055470

[11] Badiee AS Ravanshad Y Azarfar A Mehrad-Majd H Torabi S Ravanshad S. A systematic review and meta-analysis of using acupuncture and acupressure for uremic pruritus. Iran J Kidney Dis. (2018) 12:78–83. PMID: 29507269

[12] Tsai CL Lan CC Wu CW Wu YC Kuo CY Tzeng IS . Acupuncture point stimulation treatments combined with conventional treatment in chronic obstructive pulmonary disease: a systematic review and network meta-analysis. Front Med. (2021) 8:586900. doi: 10.3389/fmed.2021.586900, PMID: 34150784 PMC8211776

[13] Tang Y Cheng S Wang J Jin Y Yang H Lin Q . Acupuncture for the treatment of itch: peripheral and central mechanisms. Front Neurosci. (2021) 15:786892. doi: 10.3389/fnins.2021.786892, PMID: 35431769 PMC9005788

[14] Sterne JAC Savović J Page MJ Elbers RG Blencowe NS Boutron I . Rob 2: a revised tool for assessing risk of Bias in randomised trials. BMJ (Clinical research ed). (2019) 366:l4898. doi: 10.1136/bmj.l4898, PMID: 31462531

[15] White I . Network meta-analysis. Stata J. (2015) 15:951–85. doi: 10.1177/1536867X1501500403

[16] Carlsson C Wallengren J. Therapeutic and experimental therapeutic studies on acupuncture and itch: review of the literature. J Eur Acad Dermatol Venereol. (2010) 24:1013–6. doi: 10.1111/j.1468-3083.2010.03585.x, PMID: 20337812

[17] Liang Y Jacobi HH Reimert CM Haak-Frendscho M Marcusson JA Johansson O. Cgrp-Immunoreactive nerves in Prurigo Nodularis – an exploration of neurogenic inflammation. J Cutan Pathol. (2000) 27:359–66. doi: 10.1034/j.1600-0560.2000.027007359.x 10917163

[18] Lu P-H Chung C-H Chuo H-E Lin I-H Lu P-H. Efficacy of acupoint stimulation as a treatment for uremic pruritus: a systematic review and meta-analysis. Front Med. (2022) 9:9. doi: 10.3389/fmed.2022.1036072, PMID: 36530891 PMC9751623

[19] Lu PH Lai CC Chiu LY Wang JY Lu PH. Comparative efficacy of Chinese herbal medicines for dialysis patients with uremic pruritus: a systematic review and network meta-analysis. Front Pharmacol. (2023) 14:1064926. doi: 10.3389/fphar.2023.1064926, PMID: 36733503 PMC9886678

[20] Ardinata D Zain-Hamid R Roesyanto-Mahadi ID Mihardja H. Interleukin-31 serum and pruritus dimension after acupuncture treatment in hemodialysis patients: a randomized clinical trial. Open Access Maced J Med Sci. (2021) 9:196–201. doi: 10.3889/oamjms.2021.5599

[21] Zhang SW . Clinical observation on the acupuncture in the treatment of skin itching due to blood deficiency and wind-drying in maintenance hemodialysis (graduate thesis). Guangzhou, China: Guangzhou University of ChineseMedicine (2020).

[22] Park HJ Ahn S Lee H Hahm DH Kim K Yeom M. Acupuncture ameliorates not only atopic dermatitis-like skin inflammation but also acute and chronic serotonergic itch possibly through blockade of 5-Ht (2) and 5-Ht (7) receptors in mice. Brain Behav Immun. (2021) 93:399–408. doi: 10.1016/j.bbi.2021.01.027 33524554

[23] Wang YM Ma TM. Effect of pre-acupuncture intervention on serum Ige and cutaneous phosphorylated tyrosine-protein kinase expression in rats with Urticaria. Zhen Ci Yan Jiu. (2020) 45:111–6. doi: 10.13702/j.1000-0607.1901886, PMID: 32144920

[24] Wang J Liu B Tsai BL. Acupoint injection for uremic cutaneous pruritus in hemodialysis maintenance: 55 cases. Jiangsu J Tradit Chin Med. (2021) 53:51–4. doi: 10.19844/j.cnki.1672-397X.2021.09.019

[25] Wang Y-M Gao J-H Lu B Peng J Fan B Cui J-J . Comparison of the effects of Carbamyl-Β-Methylcholine chloride administered by intravenous, intramuscular and intra-acupuncture point injections. J Tradit Chin Med. (2012) 32:93–8. doi: 10.1016/S0254-6272(12)60039-9, PMID: 22594110

[26] Chao MT Wade CM Booth SL. Increase in plasma Phylloquinone concentrations following acupoint injection for the treatment of primary dysmenorrhea. J Acupunct Meridian Stud. (2014) 7:151–4. doi: 10.1016/j.jams.2014.01.004 24929459 PMC4096683

[27] Yang T Zhao J Guo Q Wang Y Si G. Acupoint injection treatment for non-dialysis dependent chronic kidney disease: a meta-analysis of randomized controlled trials. Medicine. (2020) 99:e23306. doi: 10.1097/MD.0000000000023306 33371063 PMC7748216

[28] Chen GM Du JT Kuang H He YC Li J. Clinical observation on treating skin itching in maintenance hemodialysis patients by self blood Acupoint injection plus point massage. Clin J Chin Med. (2017) 9:100–2. doi: 10.1007/s11726-019-1109-8

[29] Karjalian F Momennasab M Yoosefinejad AK Jahromi SE. The effect of acupressure on the severity of pruritus and laboratory parameters in patients undergoing hemodialysis: a randomized clinical trial. J Acupunct Meridian Stud. (2020) 13:117–23. doi: 10.1016/j.jams.2020.05.002 32497714

[30] Oleson T . Overview and history of Auriculotherapy. Auriculotherapy Manual: Chinese and Western Systems of Ear Acupuncture (2003). 2 p. doi: 10.1016/B978-0-7020-3572-2.00001-X

[31] Nogier PFM Petitjean F Mallard A. Points Réflexes Auriculaires: Maisonneuve (1987)

[32] Yan CN Yao WG Bao YJ Shi XJ Yu H Yin PH . Effect of auricular acupressure on uremic pruritus in patients receiving hemodialysis treatment: a randomized controlled trial. Evid Based Complement Alternat Med. (2015) 2015:593196:1–8. doi: 10.1155/2015/593196, PMID: 26495017 PMC4606162

[33] Ronco C Bellomo R. Hemoperfusion: technical aspects and state of the art. Crit Care. (2022) 26:135. doi: 10.1186/s13054-022-04009-w, PMID: 35549999 PMC9097563

[34] Chen SJ Jiang GR Shan JP Lu W Huang HD Ji G . Combination of maintenance hemodialysis with hemoperfusion: a safe and effective model of artificial kidney. Int J Artif Organs. (2011) 34:339–47. doi: 10.5301/ijao.2011.7748 21534244

[35] Zhao D Wang Y Wang Y Jiang A Cao N He Y . Randomized control study on hemoperfusion combined with hemodialysis versus standard hemodialysis: effects on middle-molecular-weight toxins and uremic pruritus. Blood Purif. (2022):1–11. doi: 10.1159/000525225, PMID: 35952629

[36] Ma LL Chang PJ Ren K. Treatment of uremic pruritus with acupuncture combined hemodialysis plus hemoperfusion. J Beijing Univ Tradit Chin Med. (2014) 21:28–30. doi: 10.3969/j.issn.2095-6606.2014.05.008

[37] Koh TJK . Nocturnal hemodialysis: improved quality of life and patient outcomes. Int J Nephrol Renovasc Dis. (2019) 12:59–68. doi: 10.2147/ijnrd.s165919, PMID: 31040710 PMC6452820

[38] Juan JJ Shuang YX Yue JL Juan Y Yan YW Yan C . Effect of hemoperfusion combined with acupuncture on pruritus in the elderly patients with uremia. Practical. Geriatrics. (2021) 35:1281–3.

[39] Zhang S. Clinical observation on the acupuncture in the treatment of skin itching due to blood deficiency and wind-drying in maintenance hemodialysis. Graduate Thesis of Guangzhou University of Chinese Medicine (2020)

[40] Nahidi Y Badiee S Torabi S Abbasi SZ Nazemian F Saki A. Acupuncture effect on pruritus in hemodialysis patients: a randomized clinical trial. Iran Red Crescent Med J. (2018) 12:65521. doi: 10.5812/ircmj.65521

[41] Chu LC Hsu WC Li CJ Huang HH Chen HL. Clinical effect for pruritus of replenishing and reducing acupuncture in maintenance hemodialysis and the influence of serum Ipth and Β2-mg. Chronic Pathematology J. (2018) 19:1763–6. doi: 10.16440/j.cnki.1674-8166.2018.12.046

[42] Pu LC . Effectiveness of the acupuncture combined hemodialysis plus hemoperfusion for uremic pruritus. For all Heath. (2017) 8:109–10.

[43] Chang KS Lei TS Ju SR Lan S Shiu HJ Fei TH . Improvement of uremic pruritus in hemodialysis with citric acid: 17 cases. J Jiangxi Univ Traditional Chin Med. (2017) 6:40–2.

[44] Chang F Chiu CL Huang HS Fang SS Shen Y. The effectiveness of treatment of uremic puritus with acupuncture combined hemodialysis plus hemoperfusion. J Practical Med. (2011) 27:1687–9.

[45] Ruei HR Lin WM Sha JP. Observation on therapeutic effect of 80 cases of uremic cutaneous pruritus treated with acupuncture. Chin Acupuncture Moxibustion. (2002) 22:235–6.

[46] Kao HM Chang WH Wang Y. Acupuncture for uremic cutaneous pruritus: 34 cases. J Tradit Chin Med. (2002) 5:312. 11977515

[47] Chen D Ouyang ZP Wen F. Clinical observation of patients with chronic kidney disease-mineral and bone abnormalities by auricular point pressing combined with nocturnal dialysis. Yunnan J Trad Chin Med Materia Med. (2020) 41:54–6.

[48] Yan C Yau WG Liu G Wang H Li J Shia M. Observation of auricular acupressure on the pruritus of the patients in maintenance hemodialysis. Chin J Integr Trad Western Nephrol. (2020) 21:512–4.

[49] Yan C Li J Gea L. The effection of the serum level of Il-6 on patients receiving hemodialysis treatment for uremic pruritus by auricular acupressure. Journal of integrated traditional and Western. J Integr Traditional Western Nephrol. (2021) 22:499–502.

[50] Rong Y Ting YW Jun CR Ni HN Mei LX. Auricular copper scarping therapy for uremic cutaneous pruritus: 50 cases. Fujian J TCM. (2021) 52:52–4.

[51] Zhai J . Observation on the effect of auricular point on skin pruritus in hemodialysis patients with uremia. Contempor Med Symp. (2021) 18:169–70.

[52] He CC Guo JJ Shia A Wu LC Jau DS Wu SC . Therapeutic effect of auricuiar acupressure on the treatment of pruritus in maintenance hemodiaiysis patients. Traditional Western Nephrol. (2018) 19:919–21.

[53] Li L Ma J. Effects of auricular points taping and pressing with nursing intervention on Esrd pruritus in maintenance hemodialysis patients. J Hubei Univ Chin Med. (2017) 19:92–4.

[54] Shr CJ Shiu C Shiu TY Tzou SL Hu PP Wang M. Clinical observation on treating MHD complications by hemoperfusion joint auricular acupressure. Clin J Chin Med. (2012) 4:7–9.

[55] Hsu MC Chen HW Hwu YJ Chanc CM Liu CF. Effects of thermal therapy on uremic pruritus and biochemical parameters in patients having haemodialysis. J Adv Nurs. (2009) 65:2397–408. doi: 10.1111/j.1365-2648.2009.05100.x, PMID: 19737321

[56] Deng HY . Observation of acupoint injection combined acupuncture in uremic cutaneous pruritus: 23 cases. World Latest Med Inf. (2017) 15:233–4.

[57] Wang M Shr CJ Hsiao HH. Observation of acupoint injection combined acupuncture in uremic cutaneous pruritus: 56 cases. Pract Clin J Integr Traditional Chin Western Med. (2004) 4:17–8.

[58] Yi JC Zheng MX. Therapeutic effect of infrared acupoint irradiation combined with local irradiation on uremic pruritus caused by maintenance hemodiaiysis. J Yichun Univ. (2018) 40:70–2.

[59] Akça NK Taşcı S. Acupressure and transcutaneous electrical acupoint stimulation for improving uremic pruritus: a randomized. Controlled Trial Altern Ther Health Med. (2016) 22:18–24. 27228268

[60] Jiu SG Li YS Li WY Jin F Miau HD. Umbilical acupoint sticking therapy combined different blood purification methods on cutaneous pruritus in hemodialysis patients. Gansu Zhongyi Xueyuan Xuebao. (2015) 32:44–7.

