# Supplementary material for: Comparative efficacy of acupuncture point stimulation treatments for dialysis patients with uremic pruritus: a systematic review and network meta-analysis
Source: Front Neurol. 2024 Mar 22;15:1342788. doi: 10.3389/fneur.2024.1342788 (PMC11003387; doi:10.3389/fneur.2024.1342788)
Supplement: Supplementary file 1 [file Table_1.docx]

**Supplementary Materials**

**Supplementary Figure 1.** **Risk of bias summary and graph of included studies**

(a) Risk of bias summary (b) Risk of bias graph

**Supplementary Figure 2. Cluster ranking plot**

**Supplementary Figure 3. Funnel plots of different acupuncture point stimulation treatments in overall effective rate and visual analogue scale**

(a) Overall effective rate (b) Visual analogue scale

**Supplementary Table 1. Search strategy**

**Supplementary Table 2. Characteristics of the selected studies**

1. Risk of bias summary

**
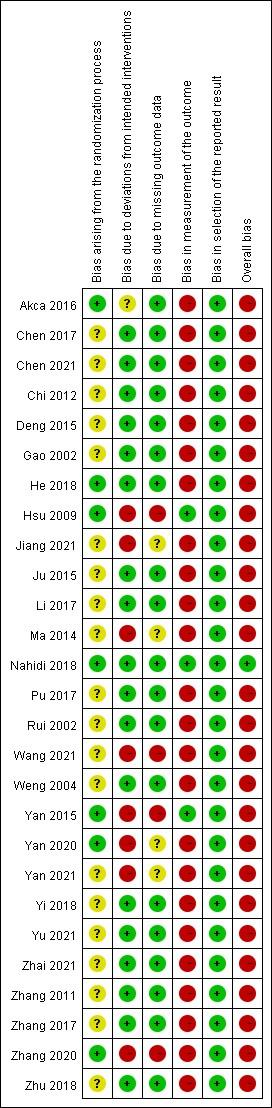
**

1. Risk of bias graph

**
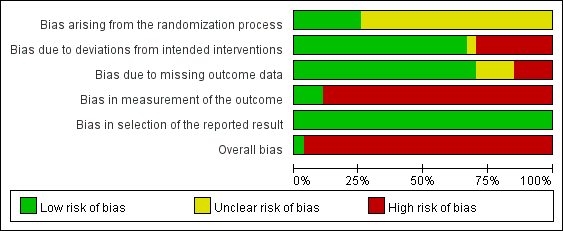
**

**Supplementary Figure 1.** Risk of bias summary and graph of included studies


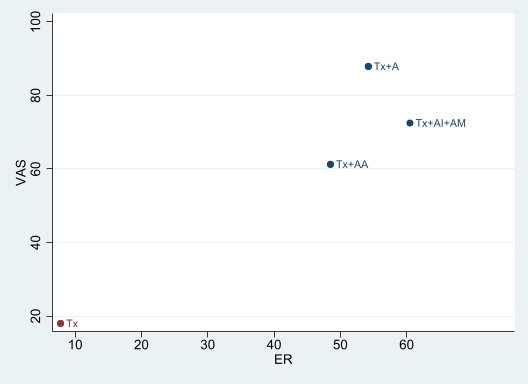


**Supplementary Figure 2. Cluster ranking plot.** The cluster ranking plot utilizes the SUCRA values as its basis. The vertical axis represents the SUCRA values for the overall ER, while the horizontal axis represents the SUCRA values for the VAS. The APSTs positioned in the upper right corner exhibit greater enhancements in both the overall ER and VAS scores than the other treatments. Abbreviations: *A*, acupuncture; *AA*, auricular acupressure; *AM*, acupoint massage; *AI*, acupoint injection; *Tx*, conventional treatment.

1. Overall effective rate


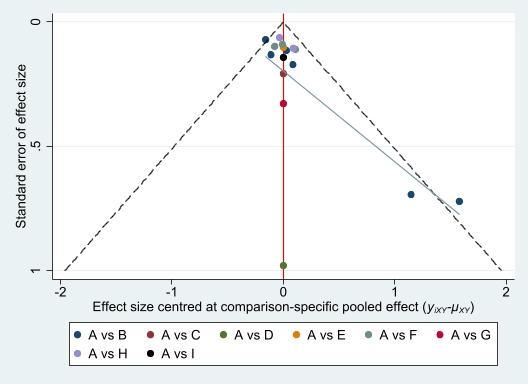


1. Visual analogue scale


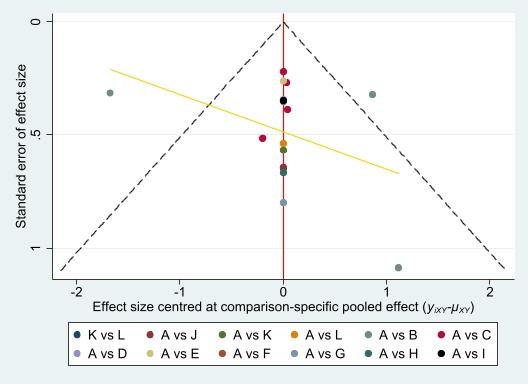


**Supplementary Figure 3.** Funnel plots of different acupuncture point stimulation treatments in overall effective rate and visual analogue scale

**Supplementary Table 1. Search strategy**

| PUBMED |
| --- |
| chronic kidney disease OR kidney injury OR kidney failure OR chronic renal failure OR end-stage renal disease OR end stage renal disease OR dialysis OR hemodialysis OR peritoneal dialysis  Uremic OR Uremia OR uremias  #1 OR #2  Pruritus OR Pruritis OR itch* OR xerosis OR skin problems OR skin disorders  Acupuncture OR Acupressure OR Shiatsu OR Zhi Ya OR Chih Ya OR Shiatzu OR auricular acupuncture OR ear acupuncture OR auricular acupressure OR ear acupressure OR auricular therapy OR auriculotherapy OR auricular needle OR otopoint OR otoneedle OR auriculoacupuncture OR otopuncture OR acupressure point OR Acupoints OR Tui Na  ((#3) AND #4 AND #5 |
| EMBASE |
| 'chronic kidney disease'/exp OR 'chronic kidney disease' OR (chronic AND ('kidney'/exp OR kidney) AND ('disease'/exp OR disease)) OR 'kidney injury'/exp OR 'kidney injury' OR (('kidney'/exp OR kidney) AND ('injury'/exp OR injury)) OR 'kidney failure'/exp OR 'kidney failure' OR (('kidney'/exp OR kidney) AND ('failure'/exp OR failure)) OR 'chronic renal failure'/exp OR 'chronic renal failure' OR (chronic AND ('renal'/exp OR renal) AND ('failure'/exp OR failure)) OR 'end-stage renal disease'/exp OR 'end-stage renal disease' OR ('end stage' AND ('renal'/exp OR renal) AND ('disease'/exp OR disease)) OR 'end stage renal disease'/exp OR 'end stage renal disease' OR (end AND stage AND ('renal'/exp OR renal) AND ('disease'/exp OR disease)) OR 'dialysis'/exp OR dialysis OR 'hemodialysis'/exp OR hemodialysis OR 'peritoneal dialysis'/exp OR 'peritoneal dialysis' OR (peritoneal AND ('dialysis'/exp OR dialysis))  uremic OR 'uremia'/exp OR uremia OR uremias  #1 OR #2  'pruritus'/exp OR pruritus OR 'pruritis'/exp OR pruritis OR itch* OR 'xerosis'/exp OR xerosis OR 'skin problems' OR (('skin'/exp OR skin) AND problems) OR 'skin disorders' OR (('skin'/exp OR skin) AND ('disorders'/exp OR disorders))  'acupuncture'/exp OR acupuncture OR 'acupressure'/exp OR acupressure OR 'shiatsu'/exp OR shiatsu OR 'zhi ya' OR 'chih ya' OR shiatzu OR 'auricular acupuncture'/exp OR 'auricular acupuncture' OR 'ear acupuncture'/exp OR 'ear acupuncture' OR 'auricular acupressure'/exp OR 'auricular acupressure' OR 'ear acupressure' OR 'auricular therapy'/exp OR 'auricular therapy' OR 'auriculotherapy'/exp OR auriculotherapy OR 'auricular needle' OR otopoint OR otoneedle OR 'auriculoacupuncture'/exp OR auriculoacupuncture OR otopuncture OR 'acupressure point' OR 'acupoints'/exp OR acupoints OR 'tui na'/exp OR 'tui na' ('formula'/exp OR formula)  #3 AND #4 AND #5 ('pill'/exp OR pill OR wan) |
| Cumulative Index to Nursing and Allied Health Literature (CINAHL) |
| (chronic kidney disease OR kidney injury OR kidney failure OR chronic renal failure OR end-stage renal disease OR end stage renal disease OR dialysis OR hemodialysis OR peritoneal dialysis)  (uremic OR Uremia OR uremias)  #1 OR #2  (Pruritus OR Pruritis OR itch* OR xerosis OR skin problems OR skin disorders)  Acupuncture OR Acupressure OR Shiatsu OR Zhi Ya OR Chih Ya OR Shiatzu OR auricular acupuncture OR ear acupuncture OR auricular acupressure OR ear acupressure OR auricular therapy OR auriculotherapy OR auricular needle OR otopoint OR otoneedle OR auriculoacupuncture OR otopuncture OR acupressure point OR Acupoints OR Tui Na  #3 AND #4 AND #5 |
| Cochrane Central Register of Controlled Trials |
| (chronic kidney disease OR kidney injury OR kidney failure OR chronic renal failure OR end-stage renal disease OR end stage renal disease OR dialysis OR hemodialysis OR peritoneal dialysis)  (uremic OR Uremia OR uremias)  #1 OR #2  (Pruritus OR Pruritis OR itch* OR xerosis OR skin problems OR skin disorders)  (Acupuncture OR Acupressure OR Shiatsu OR Zhi Ya OR Chih Ya OR Shiatzu OR auricular acupuncture OR ear acupuncture OR auricular acupressure OR ear acupressure OR auricular therapy OR auriculotherapy OR auricular needle OR otopoint OR otoneedle OR auriculoacupuncture OR otopuncture OR acupressure point OR Acupoints OR Tui Na)  #3 AND #4 AND #5 |
| Airiti library |
| chronic kidney disease OR kidney failure OR dialysis OR 透析 OR 血透 OR 腹透 OR 腎衰竭 OR 腎功能衰竭 OR 腎功能不全 OR 腎小球腎炎 OR 腎炎 OR uremic OR Uremia OR uremias OR 尿毒  pruritus OR Pruritis OR itch* OR xerosis OR Skin Diseases OR 皮膚搔癢 OR 搔癢 OR 癢  Acupuncture OR Acupressure OR 針灸 OR 針刺 OR 針 OR 穴位按摩 OR 指壓 OR 穴位 OR 穴 OR 耳穴 OR 耳針 OR 耳豆  #1 AND #2 AND #3 |
| China National Knowledge Infrastructure |
| 肾 + 尿毒 + 透析  瘙痒 + 皮肤瘙痒 + 痒  Acupuncture + Acupressure + 針灸 + 針刺 + 針 + 穴位按摩 + 指壓 + 穴位 + 穴 + 耳穴 + 耳針 + 耳豆  #1 AND #2 AND #3 |
| Wangfang |
| chronic kidney disease OR kidney failure OR dialysis OR 透析 OR 腎 OR uremic OR 尿毒  pruritus OR Pruritis OR itch* OR xerosis OR Skin Diseases OR 皮膚搔癢 OR 搔癢 OR 癢  Acupuncture OR Acupressure OR 針灸 OR 針刺 OR 針 OR 穴位按摩 OR 指壓 OR 穴位 OR 穴 OR 耳穴 OR 耳針 OR 耳豆  #1 AND #2 AND #3 |

**Supplementary Table 2. Characteristics of the selected studies**

| Study (year) | Study design | Inclusion criteria | No. of patients | Age (year-old) | Treatment (Route, dosage, and frequency) | Control  (Route, dosage, and frequency) | Concomitant treatment | Duration | Inspection data | Pruritus severity assessment tool | PS (before → after),  Experimental  Control | Effective rate | Follow up duration | Acupoint |
| --- | --- | --- | --- | --- | --- | --- | --- | --- | --- | --- | --- | --- | --- | --- |
| **Acupuncture** | | | | | | | | | | | | | |  |
| Jiang et al.(38) (2021)^†^ | RCT | HD | T1(hemoperfusion): 14  T2(hemoperfusion + A): 14  C: 14 | T1: 72.43(7.56)  T2: 73.50(7.77)  C: 72.93(9.09) | Hemoperfusion  A  (3 times/W)  HD | Placebo + HD | Conventional Tx | NA | Ca, P, iPTH, hs-CRP, IL-6 | VAS | T1:  2(3.70) → 1(3.29)  T2:  1.5(3.08) → 0(0.61)→ 0(0.61)  C: 2.00(3.70) | NA | NA | LI4  LI11  SP6  SP10  ST36 |
| Zhang et al.(39)  (2020) | RCT | HD | T: 30  C: 33 | T: 53.63(9.40)  C:  52.23(7.95) | A  (3 times/W) + HD | HD | Conventional Tx | 4W | CRP, Hb, albumin, BUN, Cr, Ca, P, PTH, Eosinophil, IgE | VAS | ΔT: 5(3-8)  ΔC: 2(-1-4) | T: 13/35  C: 2/35 | NA | LI11  SP10 SP6 |
| Nahidi et al.(40)  (2018) | RCT | HD | T: 15  C: 11 | T: 54.67(11.40)  C:  41.36(16.21 | A  (3 times/W) + HD | Placebo + HD | Conventional Tx | 6W | NA | VAS | T: 9.87(0.35) → 3.93(2.85)  C: 9.45(0.93) → 8.18(1.40) | NA | NA | SP6 SP10 LIV3 LI4 |
| Chu et al.(41) (2018) | RCT | HD | T: 20  C: 20 | T: 44.80(11.50)  C:  45.50(12.00) | A  (2 times/W) + HD | Loratidine  (10mg QD) +HD | Conventional Tx | 12W | BUN, Cr, P, PTH, β2-MG | VAS | T: 8.65(1.24) → 1.45(0.58)  C: 8.60(1.18) → 5.24(1.28) | T: 19/20  C: 13/20 | NA | SP10 ST36 SP6 LI4  LI11 DU20 |
| Pu et al.(42) (2017) | RCT | HD | T: 27  C: 27 | T: 63.52(5.18)  C:  63.42(5.32) | A  (2 times/W) + HD | HD | Conventional Tx | 10D | NA | Dirk. LKuypers scale(DLKS) | T: 25.5(5.3) → 10.4(3.4)  C: 25.2(5.1) → 18.9(4.1) | T: 24/27  C: 20/27 | NA | LI4 LI11 ST36 LU5 |
| Chang et al.(43) (2017) | RCT | HD | T1(A): 16  T2(citrate): 17  C: 17 | Total:  45.30(18.90) | T1: A + HD  T2: citrate dialysate | HD | Conventional Tx | Not mentioned | CRP, WBC | Guidelines for clinical research of Traditional Chinese Drug Research | T1: 12.12(1.78) → 9.16(1.67)  T2:  12.17(1.75) → 4.08(1.84)  C: 12.08(1.82) → 10.80(1.96) | T1: 9/16  T2: 15/17  C: 2/15 | NA | LI11  GB31  ST36  SP10  BI17 |
| Ma et al.(36) (2014) | RCT | HD | T: 23  C: 23 | T: 64.33(13.77)  C:  60.74(16.36) | A  (3 times/W) + HD | HD | Conventional Tx | 16W | NA | Clinical effect | NA | T: 18/23  C: 1/19 | NA | SP10  LI4 |
| Chang et al.(44) (2011) | RCT | HD | A+HDF: 15  HDF: 15  C(HD): 16 | A+HDF: 52.30(12.60)  HDF:  49.60(13.40)  C:  53.20(15.90) | A  (2 times/W) + HD  + HDF | HDF and  HD | Conventional Tx | 12W | P, PTH | NA | NA | A+HDF: 13/15  HDF: 10/15  C: 3/16 | NA | LI4  LI11  ST36  SP10  LU5  BI17 |
| Rui  et al.(45)  (2002) | RCT | HD | T: 80  C: 70 | T:  21-73  C:  24-69 | A  (2 times/W or 3 times/2W) + HD | Calcitrol  (2ug two times/W or 3 times/2W)  + HD | Conventional Tx | 16W | NA | NA | NA | T: 71/80  C: 62/80 | 12M | LI11  ST36  SP6  SP10 |
| Kao et al.(46)  (2002) | RCT | HD | T: 34  C: 34 | Total  22~72(mean 43.6) | A  (2 times/W) + HD | Chlorpheniramine + HD | Conventional Tx | 4W | NA | NA | NA | T: 33/34  C: 24/34 | NA | LI11  ST36 |
| **Auricular acupressure (AA)** | | | | | | | | | | | | | | |
| Chen et al. (47) (2020) | RCT | HD | AA+ND: 30  ND: 30  C: 30 | AA+ND: 44.10(1.60)  ND: 43.90(1.40)  C: 44.20(1.50) | ND  (3 times/W)  AA+ND: AA  (3~5 times/D)  + ND | HD | Conventional Tx | 48W | Ca, P, PTH, ALP, vitamin D | NA | NA | itching:  AA+ND: 20/30  ND: 8/30  C: 5/30 | NA | TF4  kidney  spleen  stomach  subcortical  sympathy |
| Yan et al.(48)  (2020) | RCT | HD | T: 39  C: 39 | T: 57.16(17.01)  C: 54.40(8.37) | AA  (9 times/2ds) + HD | HD | Conventional Tx | 12W | Ca, P, PTH, amylase, histamine, IL-2 | VAS | T: 5.84(1.82) → 3.88(1.50)  C: 5.84(2.06) → 5.37(1.90) | NA | NA | kidney  lung  heart  endocrine  subcortical  TF4 |
| Yan et al.(49) (2021) | RCT | HD | T: 32  C: 36 | T: 57.72(12.16)  C: 61.06(11.77) | AA  (9 times/2ds) + HD | HD | Conventional Tx | 12W | Ca, P, PTH, IL-2, IL-6, IL-8, IL-10 | VAS | T: 5.69(0.97) → 2.66(0.87)  C: 5.75(0.94) → 4.19(0.92) | NA | NA | kidney  lung  heart  endocrine  subcortical  TF4 |
| Yu et al.(50) (2021) | RCT | HD | T: 50  C: 50 | T: 55.60(1.90)  C: 53.60(2.50) | Auricular scraping  (1 time/W) | Loratidine 10mg/d | Conventional Tx | 4W | NA | FIIQ | T: 8.42(3.30) → 5.41(2.25)  C: 8.48(4.21) → 6.79(3.02) | T: 43/50  C: 38/50 | 8W | Lung  Large intestine  Shenmen(TF4)  Endocrine(CO18)  Adrenal gland  Occiput |
| Zhai et al.(51) (2021) | RCT | HD | T: 50  C: 50 | T: 62.98(3.65)  C: 63.23(3.92) | AA  (4 times/d) + HD | Loratadine (10mg QD) + HD | Conventional Tx | 12W | NA | PSQI | T: 20.21(2.15) → 13.67(2.97)  C: 20.34(2.72) → 17.13(2.12) | Itching:  T: 46/50  C: 38/50 | NA | kidney  spleen  stomach  sympathy  subcortical  TF4 |
| He et al.(52)  (2018) | RCT | HD | T: 34  C: 35 | T:  54.18(10.76)  C: 50.23(12.69) | AA  (4~5 times/2ds) + HD | HD | Conventional Tx | 4W | Ca, P, PTH, Cr, BUN, albumin, Hb | VAS | T: 4.59(2.03) → 1.79(1.10)  C: 3.80(1.39) → 3.29(1.13) | NA | NA | lung  endocrine  adrenal |
| Li et al.(53) (2017) | RCT | HD | T: 40  C: 40 | T:  53.80(3.60)  C: 55.10(1..80) | AA  (4 times/d) + HD | Acrivastine(8mg 3 times/d) + HD | Conventional Tx | 4W | NA | NA | NA | T: 38/40  C: 28/40 | NA | A:  kidney  heart  lung  liver  spleen  Sanjio  B:  bladder  Shenmen  HX1  HX6.7i  SF1.2i  AT2.3.4i |
| Yan et al.(32) (2015) | RCT | HD | T: 32  C: 30 | Total 20~65 | AA  (5~8 times/d)  + HD | Placebo  + HD | Conventional Tx | 6W | Ca, P, PTH, histamine, substance P, PAR-2, tryptase | VAS | T: 5.75(2.03) → 3.84(1.69)  C: 5.60(2.13) → 5.57(2.29) | NA | NA | Shenmen(TF4), kidney(CO10), lung(CO14), endocrine(CO18), subcortical(AT4) |
| Chi et al.(54) (2012) | RCT | HD | T: 30  C1(HD+HP): 30  C2(HD): 30 | Total 58.00(17.00) | AA  (>4 times/d)  + HD | HD+HP or HD | Conventional Tx | 4W | Ca, P, PTH, BUN, β2-MG, Cr | NA | NA | T: 26/29  C1: 23/27  C2: 10/28 | NA | A:  heart  lung  liver  spleen  Sanjiao  B:  bladder  Shenmen(TF4) |
| **Acupoint far infrared (AFIR)** | | | | | | | | | | | | | | |
| Hsu et al.(55) (2009) | RCT | HD | T: 21  C: 20 | T:  57.14(2.74)  C: 66.90(3.06) | AFIR  (1 time/d, 2 ds/W) | HD | Conventional Tx | 18W | Ca, P, albumin, Urea, ALK-P, Hb, PTH | VAS | T: 18.57(1.41) → 10.71(1.17)  C: 16.50(1.35) → 10.70(1.03) | NA | NA | SP6 |
| **Acupoint injection (AI)** | | | | | | | | | | | | | | |
| Wang et al.(24) (2021) | RCT | HD | T: 55  C: 54 | T:  56.40(8.60)  C: 56.40(8.80) | AI  (2 times/W)  + Neurotin  (0.1g 2 times/d)  + HD | Neurotin + HD | Conventional Tx | 4W | Ca, P, PTH | VAS | T: 6.43(1.24) → 3.35(1.52)  C: 6.25(1.22) → 4.38(1.19) | NA | NA | LI11  ST36 |
| **Acupoint injection and Acupuncture (AI + acupuncture)** | | | | | | | | | | | | | | |
| Deng et al.(56) (2017) | RCT | HD | T: 23  C: 23 | Total  42.73(3.10) | AI  (2 times/W) + acupuncture  + cetirizine  (10mg QHS) | HD | Conventional Tx | 12W | NA | NA | NA | T: 22/23  C: 19/23 | NA | AI + A:  LI11  ST36  SP10 |
| Wang et al.(57) (2004) | RCT | HD | T: 56  C: 54 | T:  29~78  C:  25~69 | AI + acupuncture  (2 times/W or 3 times/2Ws)  + HD | Calcitrol  (0.25ug 1 time/d or 1 time/2ds)  + HD | Conventional Tx | 12W | NA | NA | NA | T: 51/56  C: 48/54 | NA | A:  EM40  GB31  LI4  LI11  ST36  SP6  SP9  SP10  AI:  BI17  BI23 |
| **Acupoint injection + Acupoint massage (AI + AM)** | | | | | | | | | | | | | | |
| Chen et al.(28) (2017) | RCT | HD | T: 30  C: 30 | T:  49.17(12.20)  C: 49.77(1.24) | AI  (2~3 times/W)  + AM  (2~3 times/W) + HD | HD | Conventional Tx | 8W | NA | VAS | T: 7.50(1.11) → 5.23(1.36)  C: 7.33(1.32) → 7.27(1.36) | T: 27/30  C: 20/30 | NA | AI:  LI11  ST36  AM:  DU14  DU23  EM1  EM2  HT7  GB20  LI4  SP6  SP10  PC4 |
| **Acupoint infrared (AIR)** | | | | | | | | | | | | | | |
| Yi et al.(58) (2018) | RCT | HD | T: 20  C: 20 | T:  56.37(4.22)  C: 56.89(4.19) | AIR  (2 times/W) + HD | HD | Conventional Tx | 5W | Ca, P, PTH, albumin, BUN, Cr, Hb, WBC, Plt, | VAS | T: 7.82(1.69) → 4.17(2.86)  C: 7.49(1.30) → 6.02(2.15) | NA | NA | LI11  SP6  SP10 |
| **Acupoint massage (AM)** | | | | | | | | | | | | | | |
| Akca et al.(59) (2016) | RCT | HD | AM: 25  ATENS: 24  C: 25 | AM: 55.24(10.13)  ATENS:  48.08(9.05)  C:  45.84(10.40) | AM  (3 times/W)  ATENS  (3 times/W) | HD | Conventional Tx | 4W | NA | VAS | AM:  6.84(1.70) → 3.36(2.37)  ATENS:  7.37(1.31) → 3.12(2.15)  C:  6.92(1.41) → 5.08(1.55) | NA | NA | ATENS:  LI-11 |
| **Acupoint sticking therapy (AST)** | | | | | | | | | | | | | |  |
| Jiu et al.(60) (2015) | RCT | HD | T1(HD): 20  T2(HD+HPF): 20  T3(HD+HF): 20  C1(HD): 20  C2(HD+HPF): 20  C3(HD+HF): 20 | T1: 45.20(1.90)  T2:  44.60(2.00)  T3: 45.30(1.80)  C1: 45.30(2.10)  C2: 44.70(2.70)  C3: 45.1(2.20) | AST  (Difuzi, Tusizi, Mudanpi, and Taoren/2ds) | HD, HPF, HF | Conventional Tx | 12W | Ca, P, PTH, Hb, BUN, Cr | VAS | T1 vs C1  T1: 5.95(2.26) → 5.20(2.49)  C1: 6.65(1.57) → 5.05(1.64)  T2 vs C2  T2: 6.85(1.56) → 3.20(1.08)  C2: 6.40(1.78) → 3.75(1.12)  T3 vs C3  T3: 6.90(1.80) → 1.80(2.03)  C3: 5.85(1.89) → 2.30(2.03) | NA | NA | umbilicus |
| **Acupoint transcutaneous electrical** nerve **stimulation (ATENS)** | | | | | | | | | | | | | | |
| Akca et al.(59) (2016) | RCT | HD | AM: 25  ATENS: 24  C: 25 | AM: 55.24(10.13)  ATENS:  48.08(9.05)  C:  45.84(10.40) | AM  (3 times/W)  ATENS  (3 times/W) | HD | Conventional Tx | 4W | NA | VAS | AM:  6.84(1.70) → 3.36(2.37)  ATENS:  7.37(1.31) → 3.12(2.15)  C:  6.92(1.41) → 5.08(1.55) | NA | NA | ATENS:  LI-11 |
| RCT: randomized controlled trial, T: treatment group, C: control group, Tx: conventional treatment, NA: not applicable, HD: hemodialysis, HDF: hemodiafiltration, HF: hemofiltration, HP: hemoperfusion, ND: nocturnal dialysis, AA: auricular acupressure, ATENS: Acupoint transcutaneous electrical nerve stimulation, W: week, d: day, 5DIS: 5-D itch scale, VAS: visual analog scale, DRKS: Dirk R. Kuypers scale, TCM syndrome score: traditional Chinese medicine syndrome score, PSQI: *Pittsburgh Sleep Quality Index,* 12-PSS: 12-Item Pruritus Severity Scale, Δ: difference between two numbers, ^†^: median (interquartile range) | | | | | | | | | | | | | | |
